# A negative bias in decoding positive social cues characterizes emotion processing in patients with symptom-remitted Borderline Personality Disorder

**DOI:** 10.1186/s40479-019-0114-3

**Published:** 2019-11-15

**Authors:** Nikolaus Kleindienst, Sophie Hauschild, Lisa Liebke, Janine Thome, Katja Bertsch, Saskia Hensel, Stefanie Lis

**Affiliations:** 1 Institute of Psychiatric and Psychosomatic Psychotherapy, Central Institute of Mental Health, Medical Faculty Mannheim, Heidelberg University, PO Box 12 21 20, 68072 Mannheim, Germany; 2 Institute for Psychosocial Prevention, University Heidelberg, Heidelberg, Germany; 3 Department of Psychiatry, Western University, London, Canada; 4 Department of Theoretical Neuroscience, Central Institute of Mental Health, Medical Faculty, Heidelberg University, Mannheim, Germany; 5 Department of General Psychiatry, Center for Psychosocial Medicine, University of Heidelberg, Heidelberg, Germany; 6 Department of Psychology, LMU Munich, Munich, Germany

**Keywords:** Emotion recognition, Affiliation, Symptom-remitted BPD, Confidence, Negative bias, Social cognition, Confidence

## Abstract

**Background:**

Impairments in the domain of interpersonal functioning such as the feeling of loneliness and fear of abandonment have been associated with a negative bias during processing of social cues in Borderline Personality Disorder (BPD). Since these symptoms show low rates of remission, high rates of recurrence and are relatively resistant to treatment, in the present study we investigated whether a negative bias during social cognitive processing exists in BPD even after symptomatic remission. We focused on facial emotion recognition since it is one of the basal social-cognitive processes required for successful social interactions and building relationships.

**Methods:**

Ninety-eight female participants (46 symptom-remitted BPD [r-BPD]), 52 healthy controls [HC]) rated the intensity of anger and happiness in ambiguous (anger/happiness blends) and unambiguous (emotion/neutral blends) emotional facial expressions. Additionally, participants assessed the confidence they experienced in their own judgments.

**Results:**

R-BPD participants assessed ambiguous expressions as less happy and as more angry when the faces displayed predominantly happiness. Confidence in these judgments did not differ between groups, but confidence in judging happiness in predominantly happy faces was lower in BPD patients with a higher level of BPD psychopathology.

**Conclusions:**

Evaluating social cues that signal the willingness to affiliate is characterized by a negative bias that seems to be a trait-like feature of social cognition in BPD. In contrast, confidence in judging positive social signals seems to be a state-like feature of emotion recognition in BPD that improves with attenuation in the level of acute BPD symptoms.

## Background

Personality disorders have been defined in diagnostic classification systems as enduring and stable conditions [1, 2]. In line with this, Borderline Personality Disorder (BPD) has been regarded as a lifelong condition with severe psychopathology which is poorly ameliorated by treatment [3]. In contrast to this view, more recent studies have drawn a more optimistic picture about the long-term prognosis of BPD [4, 5]. For example, the McLean Study of Adult Development revealed cumulative rates of remission in 95% of the surviving and assessable patients over 16 years of prospective follow up [5]. Remission was here defined as no longer meeting study criteria for BPD (DSM-III-R) for a period of at least 4 years. However, these promising findings are qualified by a markedly lower cumulative rate (54%) of recovery, i.e., remission combined with the achievement of a good overall outcome indicated by having ‘at least one emotionally sustaining relationship with a close friend or life partner’ and working ‘consistently, competently and on a full-time basis’ [5, 6]. This is consistent with treatment studies revealing low levels of social and vocational functioning even after BPD-specific psychotherapeutic interventions [7–9]. Moreover, both treatment and long-term prospective studies suggest high symptom instability as indicated by unstable treatment effects at follow-up [7], as well as faster and more frequently occurring symptomatic recurrence and loss of recovery in comparison with other axis II disorders [5].

However, a disadvantageous course is not homogenously linked to all domains of symptoms, but seems to be highly variable: remission rates range between 34 and 100%, and recurrence rates between 19 and 96% can be observed depending on the single symptoms [10]. This led Zanarini et al. [10, 11] to postulate two symptom clusters linked to different time courses: ‘acute’ symptoms with a strong tendency for remission (e.g., self-mutilation, affective instability and stormy relationships) and ‘temperamental’ symptoms with lower remission and higher recurrence rates (e.g., chronic loneliness, abandonment concerns and anger). Better prognosis for the acute and more clinically urgent symptoms is promising and might be related to these symptoms being target symptoms in BPD-specific therapeutic interventions [10]. In contrast, less focus has been placed on temperamental symptoms during interventions, which may be one reason for the current impression of these symptoms being treatment resistant. Zanarini et al. [10] described two options in dealing with these symptoms: namely, helping patients to accept these feelings as encouraged in the current version of dialectical behavioural therapy, or to develop interventions tailored to specifically attenuating these symptoms. However, to develop novel treatments requires first understanding of the underlying mechanisms.

Temperamental symptoms such as fear of abandonment and loneliness have been associated with alterations in social cognitive processes such as facial emotion processing and evaluating social participation in individuals with a current diagnosis of BPD [12–17]. Facial emotion recognition is a basal process required for successful social interaction which has been shown to differentially influence interactive behaviour in healthy participants and BPD patients [18, 19]. Furthermore, it is one of the best-studied domains of social cognition in BPD (for review, see [20–22]). While several studies on facial emotion recognition in BPD suggest hypersensitivity to threat (e.g. [23–26]), recent research found that this negative bias comprises hyposensitivity to positive social cues that signal a willingness to affiliate, for example, to faces expressing positive emotions such as happiness (e.g. [16, 27, 28]). Moreover, alterations in processing ambiguous facial expression are revealed as negative evaluation of facial stimuli displaying predominantly positive facial features [24]. Beyond alterations in recognizing a specific emotion, several studies have additionally shown that BPD patients are less confident during social judgements [16, 29, 30] and that patients who were the least confident in judging less intensively happy faces felt the most lonely [16]. In general, people avoid situations that require abilities they feel less confident in [31]. As a result, low confidence in judging positive social cues in BPD may promote avoiding social situations well-suited to forming close relationships thus adding to the persistence of ‘temperamental’ symptoms such as chronic loneliness.

In summary, these findings raise the question of whether impairments in social cognitive processing of positive social cues characterize social-cognitive processing in BPD even after symptom-remission. So far, experimental studies on facial emotion processing in symptom-remitted BPD patients are sparse. One recent study used a forced-choice task to investigate how symptom-remitted BPD patients categorize ambiguous emotional faces that were blends of angry and happy facial features [32]. While the patients categorized the stimuli in a manner comparable to healthy participants, both processing times and the P300 component of event-related potentials indicated alterations during the evaluation of facial stimuli with predominantly positive facial features. These findings revealed initial evidence for alterations in emotion-recognition after symptom remission suggesting higher uncertainty during the processing of social cues that may signal another person’s willingness to form a positive social relationship. Additionally, they emphasize that the deficits are even more subtle compared with current BPD [22] and require a fine-grained methodological approach to be detected.

In the present study, we investigated facial emotional recognition in symptom-remitted BPD patients to contribute to understanding the mechanism underlying the persistence of ‘temperamental’ symptoms such as chronic loneliness and abandonment concerns. To uncover subtle impairments we chose a quantitative methodological approach to study facial emotion recognition: Instead of asking participants to categorize emotional expressions based on predefined emotion categories, participants had to assess the intensity of different emotions expressed in the same faces. Matzke, Herpertz, Berger, Fleischer, and Domes [33] found this approach to be more sensitive to subtle deficits in patients with a current BPD diagnosis compared with forced-choice tasks: they identified altered performance in BPD patients in intensity ratings, but not in accuracy of categorizing expressed emotions. In addition to the ability of emotion intensity ratings to capture subtle impairments, intensity ratings take into account that individuals tend to attribute multiple basic emotions to the same facial expression [34]. Moreover, this approach to assess emotion recognition abilities allows differentiation of dysfunctions in recognizing specific emotions from response biases, that is favouring the selection of one emotion category above others, independent of the target’s features [16, 34, 35]. For example, a bias toward attributing anger should result in higher ratings of anger across different experimental conditions. This is of particular importance in case of ambiguity when stimuli display features of multiple emotions [34]. We hypothesized that 1) symptom-remitted BPD patients show a negative bias indicated by subtle alterations in the evaluation of positive emotional facial expressions. Additionally, we investigated the participants’ confidence during the emotion intensity judgements. We hypothesized that 2) symptom-remitted BPD patients are less confident in their assessments of facial stimuli and that this effect is pronounced for positive facial expressions. Finally, we hypothesized that 3) the negative bias, i.e. alterations during the processing of positive stimuli, is stronger in those participants who report a higher level of subclinical BPD symptoms.

## Methods

### Sample

A total of 98 female subjects (age 22–46 years) participated in the study, 46 of whom were individuals with symptom-remitted BPD (r-BPD) and 52 who were healthy controls (HC) matched for age and education (See Table 1). Patients were recruited through the Clinical Research Unit on BPD funded by the German Research Foundation (DFG; KFO 256) dedicated to investigating mechanisms of disturbed emotion processing in BPD [36]. The study was conducted in accordance with the Declaration of Helsinki and was approved by the Research Ethics Board of the University of Heidelberg. Subjects provided written informed consent prior to study participation. Please note that findings for a subsample of HCs have been reported previously [16].

**Table 1:** Sample characteristics in healthy controls (HC) and symptom-remitted BPD (r-BPD)

	HC			r-BPD				
	AM		SD	AM		SD	Statistic	p
Age^a^	28.6	±	6.2	30.0	±	4.7	−1.3	.211
Years of education^b^	12.1	±	1.4	11.7	±	1.6	1083	.347
Raven^a^	53.92	±	3.90	53.31	±	4.27	0.7	.472
GAF^a^	90.29	±	6.79	71.61	±	7.60	12.7	<.001
BSL-23^a^	0.09	±	0.11	0.64	±	0.56	−6.6	<.001
BDI^a^	2.52	±	3.02	10.42	±	7.52	−6.7	<.001
RSQ^a^	4.80	±	2.88	11.96	±	5.47	−6.9	<.001

^a^ t-Test, ^b^ Mann-Whitney-U-test

Remitted BPD patients fulfilled no more than three criteria for BPD according to the DSM-IV at the time of testing and during a period of at least 2 years prior to testing. However, they had met at least five criteria at an earlier time for at least 5 years. Criteria for BPD diagnosis were assessed by trained clinical psychologists using the International Personality Disorder Examination (IPDE [37];). Axis I disorders were assessed using the Structured Clinical Interview for DSM-IV (SCID-I [38];).

General exclusion criteria were a lifetime history of psychotic or bipolar I disorder, current substance addiction, current pregnancy, history of organic brain disease, skull or brain damage, severe neurological illness or psychotropic medication at the time of testing and a positive urine toxicology screen for illicit drugs. Additional exclusion criteria for the HC group were any lifetime or current psychiatric diagnoses.

Psychopathology was assessed by self-report questionnaires: BPD symptom severity using the short version of the Borderline Symptom List (BSL-23 [39];) and depressive symptoms using the Beck Depression Inventory (BDI [40];). Rejection sensitivity was measured with a German version of the Rejection Sensitivity Questionnaire (RSQ) for adults [41, 42]. Additionally, we estimated IQ using the Raven Test (Standard Progressive Matrices [43]; and global functioning using the Global Assessment of Functioning (GAF [1];).

Detailed sample description is reported in Table 1. Of the nine DSM-IV criteria for BPD, 50% of r-BPD subjects met no DSM criterion of BPD, while 22.7% met one, 15.9% met two and 11.4% met three criteria. In more detail, 8.7% fulfilled the criterion 1 ‘Frantic efforts to avoid abandonment’, 13% criterion 2 ‘unstable, intense interpersonal relationships’, 8.7% criterion 3 ‘identity disturbance’, 6.5% criterion 4 ‘impulsivity’, 2.2% criterion 5 ‘recurrent suicidal behaviour’, 19.6% criterion 6 ‘affective instability’, 4.3% criterion 7 ‘chronic feelings of emptiness’, 8.7% criterion 8 ‘inappropriate, intense anger’, and 10.9% criterion 9 ‘paranoid ideation or dissociative symptoms’.

Of the enrolled r-BPD subjects, 30.4% fulfilled the criteria of at least one current axis-I disorder (7% mood disorders, 18% anxiety disorder, 4% eating disorders, 7% substance use disorders, 4% somatoform disorders, 2% PTSD, and 2% other disorders).

It seems worthwhile to emphasize that 88.6% of the enrolled r-BPD sample achieved GAF scores above 60 (AM = 71.6). 75% of HC and 69.6% of r-BPD participants were living together with a romantic partner or close friend (*χ*^*2*^ = 0.36, *df* = 1, *p* = .548). 94% of HC and 84.8% of r-BPD participants were employed at the time of testing (*χ*^*2*^ = 2.38, *df* = 1, *p* = .123). This suggests not only remission, but also recovery in a high percentage of the r-BPD subjects if recovery is defined as the existence of good social and vocational functioning at the time of testing (see criteria by [5]).

### Experimental tasks

All participants performed ratings of the intensity of anger and happiness in morphed facial stimuli. Each emotion intensity rating was followed by a rating of how confident participants felt in their own judgment.

Emotional facial expressions were presented on a computer screen and subjects had to assess how intensely the face expressed either anger or happiness in separate trials for each facial expression. Following each intensity rating, subjects had to assess how confident she was about this rating. Ratings were done using a 6-point scale ranging from 1 (not at all) to 6 (very strong). Trials were self-paced with forced responses: participants signalled the start of a trial by moving a cursor with pen movements on a graphic tablet to a start button displayed on the screen. Six target buttons were displayed in equal distance and semi-circular arrangement from the start button Participants indicated their rating by moving the cursor from the starting button to one of the target buttons. Stimulus presentation was ended after the participant had indicated her choice.

Facial stimuli consisted of seven different emotional expressions of six different identities (50% male, 50% female, NimStim-Face dataset [44], for information on building the morphed stimuli see [24]). Emotional expressions were ambiguous faces formed by blends of angry and happy expressions and unambiguous expressions displaying anger and happiness with low intensity. The ambiguous face stimuli were formed by blending pictures of angry and happy facial expressions at three different ratios: 60/40%, 50/50%, or 40/60% of anger and happiness, respectively. Therefore, these three types of stimuli differed in the predominance of one emotion above the other. For the unambiguous facial stimuli pictures of neutral expressions were blended with pictures of an emotional expression (happiness or anger) at a ratio of 60/40% and 50/50% (neutral/emotion) to form two types of low intense emotional facial expressions.

### Measurement variables and statistical analysis

Rating scores of emotion intensity and of confidence were analysed separately for the emotion/emotion blends and the neutral/emotion blends, using analyses of variance as omnibus tests to control for multiple testing. The emotion/emotion blends were analysed using a 2 × 3 × 2 rm-ANOVA with the independent factor of group (HC vs. r-BPD) and the repeated measurement factors of emotion type (‘blend’: anger/happiness: 60/40%, 50/50%, and 40/60%) and the emotion to be assessed (‘rating’: anger vs. happiness). The neutral/emotion blends were analysed using a 2 × 2 × 2 × 2 rm-ANOVA with the independent factor of group (HC vs. r-BPD) and the repeated measurement factors of emotion type (‘blend’: anger vs. happiness), intensity of the emotion (low (60/40%) vs. high (50/50%)), and the emotion to be assessed (‘rating’: anger vs. happiness). Degrees of freedom were corrected according to Greenhouse and Geisser if appropriate. Post hoc analysis was done with pairwise comparisons (Bonferroni-corrected for multiple testing).

To analyse whether alterations in intensity ratings and confidence in BPD are linked to BPD symptom severity, we calculated Pearson’s correlation coefficients of alterations in emotion intensity and confidence ratings with the BSL-score**.**

## Results

### Emotion intensity ratings

**Fig. 1:**
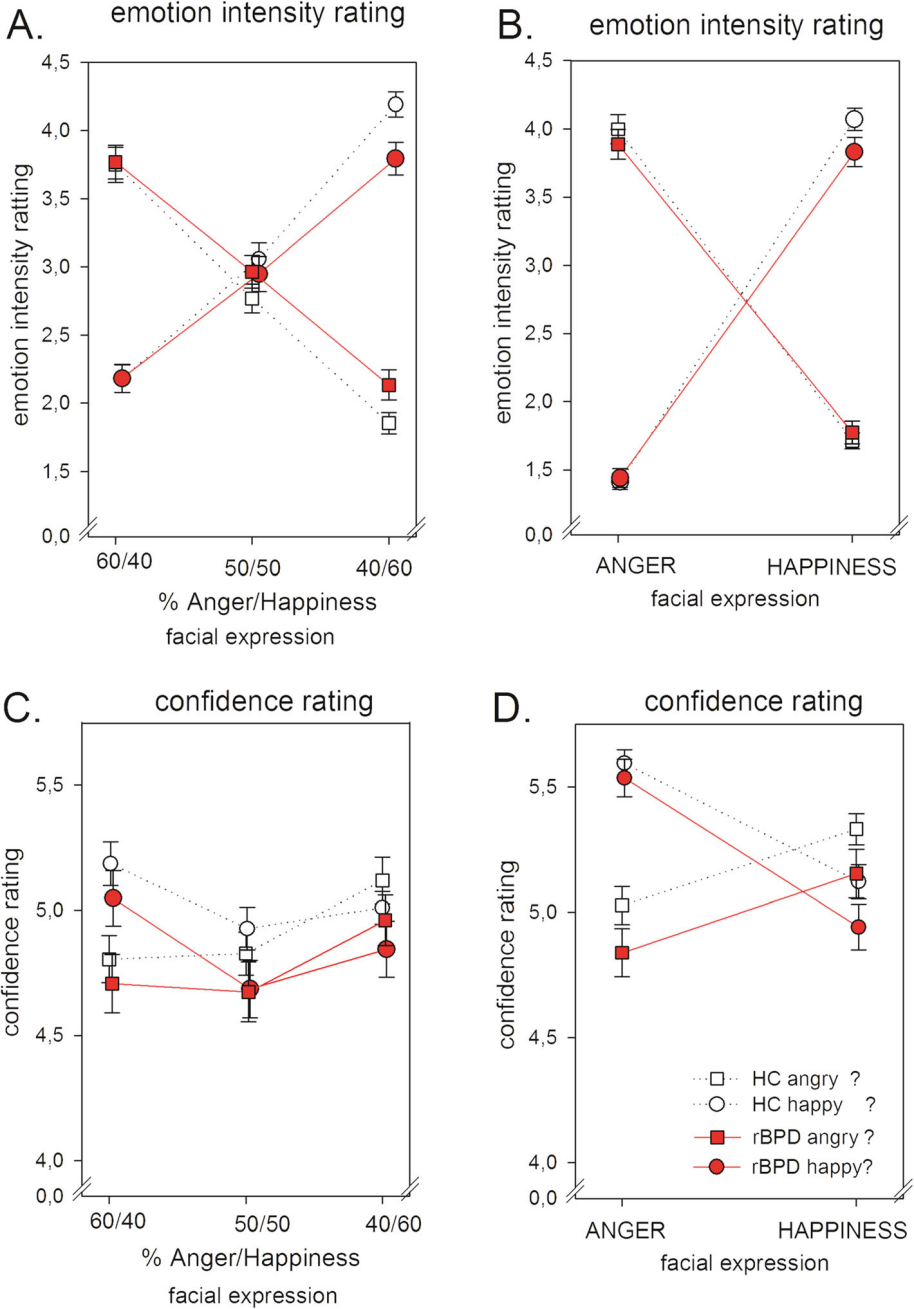
Rating scores by healthy controls (HC, unfilled symbols, dotted line) and symptom-remitted BPD subjects (r-BPD, filled symbols, solid line). **a**: Rating of the emotional intensity of anger/happiness blends. **b**. Rating of the emotional intensity of neutral/emotion blends. **c**: Rating of confidence in judging anger/happiness blends. **d**: Rating of confidence in judgment of neutral/emotion blends

When evaluating the intensity of the expressed facial emotion in ambiguous blends of happy and angry expressions, ratings differed between r-BPD subjects and HC participants depending on the presented blend and the type of the required rating (“group” x “rating” x “blend”: *F*(2,192) = 5.31, *p* = .008*, η*_*p*_^*2*^ = .052, see Fig. 1, Table 2): r-BPD subjects assessed predominantly happy blends as less happy (*p =* .010) and as more angry (*p =* .040), while no differences were observed for the evaluation of the other anger/happiness blends (all other *p >* .2). See Fig. 1a. For further details see Table 2.

**Table 2:** Results of the ANOVA of the dependent variables “emotion intensity” and “confidence rating” in stimuli with neutral/emotion and anger/happiness blends

	Emotion Intensity			Confidence		
	F	p	η_p_^2^	F	p	η_p_^2^
Anger/Happiness blends						
Group^a^	<.1	.975	<.001	1.9	.168	.020
Rating^a^	2.5	.120	.025	8.5	.005	.081
Group x rating^a^	2.0	.163	.020	.4	.516	.004
Blend^b^	1.3	.280	.013	9.2	<.001	.088
Group x blend^b^	1.0	.361	.010	.3	.712	.003
Rating x blend^b^	619.3	< .001	.866	19.6	<.001	.169
Group x rating x blend^b^	5.3	.008	.052	.1	.863	.001
Neutral/Emotion blends						
Group^a^	0.7	.417	.007	2.9	.090	.030
Rating^a^	6.2	.014	.061	63.4	<.001	.398
Group x rating^a^	0.4	.510	.005	1.4	.235	.015
Blend^a^	17.3	<.001	.153	6.2	.015	.061
Group x blend^a^	0.4	.554	.004	0.4	.534	.004
Intensity^a^	36.0	<.001	.273	18.1	<.001	.159
Group x intensity^a^	< 0.1	.930	<.001	< 0.1	.839	<.001
Rating x blend^a^	1709	<.001	.947	119.8	<.001	.555
Group x rating x blend^a^	3.7	.058	.037	0.8	.387	.008
Rating x intensity	9.0	.003	.086	1.2	.273	.012
Group x rating x intensity^a^	2.5	.120	.025	< 0.1	.870	<.001
Blend x intensity^a^	< 0.1	.908	<.001	10.8	.001	.101
Group x blend x intensity^a^	0.6	.424	.007	0.1	.747	.001
Rating x blend x intensity^a^	208.1	<.001	.684	1.7	.202	.017
Group x rating x blend x intensity^a^	0.7	.418	.007	0.6	.434	.006

^a^: *df1* = 1, *df2* = 96; ^b^: Unadjusted degrees of freedom: *df1* = 2, *df2* = 192; *p*-value adjusted according to the correction of Greenhouse & Geisser

When evaluating the intensity of the expressed facial emotion in neutral/emotion blends, r-BPD subjects assessed as a trend neutral/happy blends as less happy compared with HC subjects (“group” x “rating” x “blend”: *F*(1,102) =3.69, *p* = .058, *η*_*p*_^*2*^ *=* .037*,* post hoc comparison for the rating of happiness in neutral/happy blends: *p =* .080, all other *p* > .47 for details see Fig. 1b).

### Confidence ratings

There were no significant differences between groups in the level of confidence during judgments. However, r-BPD subjects were as a trend less confident during judgements of neutral/emotion blends compared with HC (*F*(1,96) = 2.93, *p* = .090, *η*_*p*_^*2*^ = .030 for details see Fig. 1c, d and Table 2).

### Correlations with BPD symptom severity

Correlational analyses revealed no significant correlations between alterations in the rating of the intensity of an emotional expression and BSL scores (all *p* > .05). In contrast, confidence was lower in those BPD subjects who reported higher BSL scores (during the evaluation of emotion/emotion blends: *r* = −.301, *p* = .042; neutral/emotion blends: *r* = −.297*, p* = .045). Explorative analyses of the correlations between BSL scores and confidence in the single experimental conditions revealed a correlation coefficient that would survive a Bonferroni correction for multiple testing only for the assessments of happiness in positive faces (ambiguous faces with a predominance of happiness *r* = −.452, *p* = .002; low intense happy faces *r* = −.420, *p* = .004).

## Discussion

This study investigated whether alterations in facial emotion recognition exist in symptom-remitted BPD. Our findings revealed a negative bias in judging positive facial expressions. The strength of the negative bias was not associated with the level of BPD psychopathology. Moreover, we found no differences between r-BPD and HC subjects in the confidence participants experienced in their judgements. However, a higher level of psychopathology was linked to lower confidence.

By using a quantitative approach to measure emotion recognition in symptom-remitted BPD patients, we identified subtle impairments in the evaluation of positive facial stimuli. Alterations were particularly prominent in the case of ambiguous stimuli when the emotional expressions provided both features of negative and positive emotional expressions. For these facial expressions, the r-BPD participants’ evaluations reflected a negative bias: there was both an attenuated attribution of happiness and a stronger attribution of anger to the facial expression. In line with these results, remitted BPD patients assessed as a trend unambiguous, low intense happy faces as less happy. In summary, these findings suggest that in r-BPD participants recognizing positive social cues is particularly hampered in interpersonal situations with a high level of uncertainty due to ambiguity of the available information. The confinement of impairments to the evaluation of positive cues agrees with previous findings in current and symptom-remitted BPD in studies using identical or different methodological approaches to study facial emotion recognition (e.g. [27, 45, 16]). Together with similar findings on the assessment of social scenes [28] and social belonging [12, 13, 46], emotion processing in BPD seems to be particularly impaired for social cues that may signal the willingness of social counterparts to affiliate [47]. Our data suggest that these alterations still exist after a remission from acute BPD symptoms, even if individuals achieve relatively high levels of social and vocational functioning. Our findings are in line with those reported by Schneider et al. [32] in symptom-remitted BPD and support the assumption that subtle impairments in the recognition of positive social cues constitute a trait-like feature in BPD.

In contrast to our hypotheses, our data did not reveal lower confidence during facial emotion recognition in the group of symptom-remitted BPD patients in comparison with healthy controls. In general, the remitted BPD subjects’ confidence in their judgments is well justified: they assessed the social cues for many of the experimental conditions in the same manner as healthy participants and were able to adjust their confidence depending on the targets’ features and varying difficulty to judge the intensity of an emotion across different experimental conditions [48]. Nevertheless, the BPD patients also felt confident in their negatively biased judgements when evaluating positive social cues. In general, confidence in one’s own judgments and behaviours is desirable since a lack of the latter results in negative affect and the withdrawal from domains of daily life that require skills which people feel less confident in [31]. However, feeling confident about a negative biased judgement may prevent taking a possible misinterpretation of social signals into account. Particularly misjudging positive social cues may interfere with approaching others willing to build a positive relationship. Consequently, our findings emphasize that therapeutic interventions should aim at correcting the bias in judging positive cues from a social counterpart to foster the ability to experience satisfaction with social relationships and develop a sense of belonging.

In summary, our data suggest that even after symptomatic remission, impairments in recognizing positive emotional states of others exist in BPD. One may hypothesize that these alterations in the processing of social cues relevant for forming affiliations with others may constitute a trait-like feature of BPD. This is supported by findings that in both remitted and current BPD, the strength of these alterations were neither linked to the severity of psychopathology nor comorbid disorders: Thome et al. [16] showed that neither a comorbid affective disorder nor a posttraumatic stress disorder explained the findings. In the present study, exploratory analyses of a subsample of 32 remitted BPD participants without any comorbid disorders revealed the same findings as described for the total sample (data not shown here). In contrast to the trait-like character of alterations in emotion recognition, reduced confidence in their own judgements seems to be a state-like feature of BPD depending on the psychopathological state. In the present study, reduced confidence could not be shown for the group of remitted BPD patients. Nevertheless, those patients with more severe remaining BPD symptoms felt less confident about their judgments. Additionally, exploratory correlation analyses revealed that this association was particularly strong when participants had to assess happiness in positive facial expressions. Consistent with this, reduced confidence was observed in several studies using different experimental approaches in patients with a current BPD diagnosis [16, 29, 30], but see also for divergent findings [49, 50].

Some limitations of the present study must be mentioned. Most importantly, this was a cross-sectional study and thus provides only initial hints at the development of impairments in social cognitive processes over the course of BPD. Prospective studies are required to replicate the described impairments in emotion processing and to explore whether these impairments are actually linked to a remission and recovery from BPD symptoms. Moreover, longitudinal data may also allow investigating whether the nature and strength of alterations in social cognitive processes during remission contribute to predicting symptom recurrence and loss of recovery. Thus far, research has identified several factors as predictors for a beneficial course of the disorder including no prior psychiatric hospitalizations, higher IQ, good full-time vocational record in 2 years prior to index admission, absence of an anxious cluster personality disorder, high extraversion, and high agreeableness [6]. A promising research topic for future studies is to test whether alterations in social cognition may complement these factors when predicting the prognosis of BPD. In this context, it is important to study whether the change in clusters of symptoms are indeed related to a specific intervention, or whether they may improve of their own accord or even as part of the natural time course of maturing. One may argue that the additional inclusion of a group of participants with current BPD diagnosis might have allowed a direct comparison between BPD patients with current and remitted symptoms. However, a between-subjects design would not have resolved potential issues caused by sample selection effects or confounding factors such as a higher frequency of comorbid disorders in current BPD. Moreover, further studies are needed to investigate determinants of this altered strategy of emotional face processing to identify where in the process of face evaluation the difference compared to healthy individuals is located. Finally, it must be mentioned that the generalizability of our findings is restricted, since we included only female, but no male participants. Additionally, the specificity of our findings for symptom-remitted BPD must be investigated in future studies with control groups consisting of patients in symptomatic remission from other mental disorders.

## Conclusions

In conclusion, our findings reveal alterations in facial emotion recognition in individuals with symptom-remitted BPD as one example for a social cognitive process that may be linked to the persistence of temperamental symptoms such as chronic loneliness or abandonment concern following remission of BPD symptoms. Further research on alterations in social cognition during remission seems a promising avenue to gain further insight into the mechanism underlying the high fluidity that characterizes the course of BPD, that is the change between recovery and recurrence of symptoms over time [10]. Moreover, it may contribute to the development of treatments that improve even the more persistent components of BPD psychopathology.

## Data Availability

The dataset analysed during the current study are available from the corresponding author on reasonable request.

## Abbreviations

AM: Arithmetic mean
BDI: Beck Depression Inventory
BPD: Borderline Personality Disorder
BSL-23: Borderline Symptom List (short version)
df: Degrees of freedom
DSM-5: Diagnostic and Statistical Manual, 5th edition
DSM-IV: Diagnostic and Statistical Manual, 4th edition
GAF: Global Assessment of Functioning
HC: Healthy controls
IPDE: International Personality Disorder Examination
IQ: Intelligence quotient
PTSD: Posttraumatic Stress Disorder
r-BPD: Remitted Borderline Personality Disorder
rmANOVA: Repeated measures analysis of variance
RSQ: Rejection Sensitivity Questionnaire
SCID-I: Structured Clinical Interview for DSM-IV
SD: Standard deviation
